# Therapeutic response to benzodiazepine in panic disorder subtypes

**DOI:** 10.1590/S1516-31802003000200009

**Published:** 2003-03-05

**Authors:** Alexandre Martins Valença, Antonio Egidio Nardi, Marco André Mezzasalma, Isabella Nascimento, Walter Araújo Zin, Fabiana Leão Lopes, Marcio Versiani

**Keywords:** Treatment, Panic, Disorder, Subtype, Respiration, Clonazepam, Tratamento, Subtipo, Transtorno, Pânico, Respiração, Clonazepam

## Abstract

**CONTEXT:**

This study makes a comparison between two subtypes of panic disorder regarding the clinical efficacy of clonazepam, a benzodiazepine.

**OBJECTIVES:**

To evaluate the clinical efficacy of clonazepam in a fixed dosage (2 mg/day), compared to placebo, in the treatment of panic disorder patients and to verify whether there are any differences in the responses to clonazepam between panic disorder patients with the respiratory and non-respiratory subtypes.

**TYPE OF STUDY:**

Randomized study with clonazepam and placebo.

**SETTING:**

Outpatient Anxiety and Depression Unit of the Institute of Psychiatry, Universidade Federal do Rio de Janeiro, Rio de Janeiro, Brazil.

**PARTICIPANTS:**

34 patients with a diagnosis of panic disorder with agoraphobia, between 18 and 55 years old.

**PROCEDURES:**

Administration of clonazepam or placebo for 6 weeks, in panic disorder patients, after they were classified within two subtypes of panic disorder: respiratory and non-respiratory.

**MAIN MEASUREMENTS:**

Changes in the number of panic attacks in comparison with the period before the beginning of the study; Hamilton Anxiety Scale; Global Clinical Impression Scale; and Patient's Global Impression scale.

**RESULTS:**

In the group that received clonazepam, by the end of the 6 week there was a statistically significant clinical improvement, shown by the remission of panic attacks (p < 0.001) and decrease in anxiety (p = 0.024). In the group that received clonazepam there was no significant difference between the respiratory and non-respiratory subtypes of panic disorder, regarding the therapeutic response to clonazepam.

**CONCLUSION:**

Clonazepam was equally effective in the treatment of the respiratory and non-respiratory subtypes of panic disorder, suggesting there is no difference in the therapeutic response between the two subtypes.

## RESUMO

**CONTEXTO:**

Este estudo realiza uma comparação entre dois subtipos de transtorno de pânico, em relação à eficácia clínica do clonazepam, um benzodiazepínico.

**OBJETIVO:**

Avaliar a eficácia clínica do clonazepam, em dose fixa (2 mg/dia), comparado ao placebo, no tratamento de pacientes com transtorno de pânico e verificar se há diferenças na resposta ao clonazepam em pacientes com transtorno de pânico de subtipos respiratório e não-respiratório.

**TIPO DE ESTUDO:**

Randomizado com clonazepam e placebo.

**LOCAL:**

Unidade Ambulatorial de Ansiedade e Depressão do Instituto de Psiquiatria, Universidade Federal do Rio de Janeiro, Rio de Janeiro, Brasil.

**PARTICIPANTES:**

34 pacientes com diagnóstico de transtorno de pânico com agorafobia, com idade entre 18 e 55 anos.

**PROCEDIMENTOS:**

Administração de clonazepam ou placebo por seis semanas, em pacientes com transtorno de pânico, após sua classificação em dois subtipos de transtorno de pânico: respiratório e não-respiratório.

**VARIÁVEIS ESTUDADAS:**

Mudança do número de ataques de pânico em relação ao período anterior ao tratamento; Escala de Hamilton de ansiedade; Escala de Impressão Clínica Global e Escala de Impressão Global do Paciente.

**RESULTADOS:**

No grupo que recebeu clonazepam, ao final da sexta semana houve melhora clínica estatisticamente significativa, evidenciada pela remissão de ataques de pânico (p < 0,001) e redução da ansiedade (p = 0,024). Não houve diferenças significativas em relação à resposta terapêutica ao psicofármaco, nos subtipos respiratório e não-respiratório de transtorno de pânico..

**CONCLUSÃO:**

O clonazepam foi igualmente eficaz no tratamento de pacientes com subtipos respiratório e não-respiratório de transtorno de pânico, o que sugere não haver diferença quanto à resposta terapêutica entre os subtipos.

## INTRODUCTION

Since Klein's^1^ first report showing the efficacy of imipramine in panic disorders, various studies have shown the efficacy of different drugs in the treatment of panic disorder,^2^ including the benzodiazepines.^3,5^ Chouinard et al.^6^ were the first to describe the role of clonazepam as an antipanic agent.

According to Beaudry et al.^7^ and Pollack,^8^ clonazepam is a high potency benzodiazepine with a high affinity for central benzodiazepine receptors. Its effect on the serotoninergic system may have a role in its antipanic effect. However, the exact action site through which benzodiazepines achieve the antipanic effect is still unknown.^9^

Clonazepam has been considered effective according to various studies. However, there are no studies evaluating its efficacy in different panic disorder subtypes. Most of these studies compare the efficacy of clonazepam with the efficacy of imipramine or alprazolam, since these latter two drugs had their antipanic properties discovered earlier. Tesar et al.^10^ conducted a double-blind study with alprazolam, clonazepam and placebo. They selected 44 patients with panic disorder (Diagnostic and Stastitical Manual of Metal Disorders, Third Edition, Revised — DSM-III-R). From these, 15 used clonazepam, 14 alprazolam and 15 placebo, during a 6-week period. There was a significant reduction in anxiety and panic attacks in both groups that used benzodiazepines, in comparison with the group that used placebo. The results achieved with the two benzodiazepines were considered similar: clonazepam was considered better than placebo and as effective as alprazolam.

Svebak et al.^11^ conducted a double-blind study with imipramine and clonazepam in 12 patients with panic disorder (DSM-III-R). Six patients had imipramine (mean doses from 25 to 75mg/day) and the other six had clonazepam. By the end of the fifth week of the study there was a statistically significant decrease in panic attacks and anxiety in both groups. The favorable clinical responses were maintained until the end of the study, after six months. The authors considered clonazepam to be an effective therapeutic alternative.

Panic disorder subtypes have been described in the literature.^12,14^ Briggs et al.^12^ studied the description of the last and most severe panic attack of 1,108 panic disorder patients, which were divided into two groups according to the presence or absence of conspicuous respiratory symptoms. They found that the group which had conspicuous respiratory symptoms had more spontaneous panic attacks and better responses to imipramine, while the patients from the non-respiratory subgroup had more situational panic attacks and better responses with alprazolam.

There is no trial comparing the clinical response of benzodiazepine in panic disorder subtypes. Our aim with this study was to evaluate whether there are differences in the responses to clonazepam (2mg/day) in panic disorder patients from the respiratory and non-respiratory subtypes.

## METHODS

We selected 34 patients with a diagnosis of panic disorder with agoraphobia from the Laboratory of Panic and Respiration of the Universidade Federal do Rio de Janeiro. The diagnosis was confirmed with the Structured Clinical Interview Diagnosis (SCID 1)^15^ for DSM-IV.^16^ All patients signed an informed consent form to join the study. The protocol for the study was approved by our local Ethics Committee and it was conducted following all the principles from the Helsinki Declaration.

To be enrolled in the study, all patients needed to be between 18 and 55 years old and also to have had at least three panic attacks during the two-week period before their first testing. Patients were not included if there was a previous history of cardiovascular or respiratory disease. Other conditions among the exclusion criteria were: epilepsy, pregnancy, bipolar mood disorder, psychotic symptoms, mental retardation and current major depression. All patients also had to be free of psychotropic drugs for at least one week prior to the study, and have one urinary test excluding the presence of benzodiazepines and other medications.

The patients were randomly selected to take either clonazepam (2 mg/day) or placebo, over a six-week period (the total duration of the study). The clinical symptoms of the most severe and recent panic attack were evaluated for each patient before the beginning of the study. The patients were diagnosed within the respiratory or non-respiratory subtypes, according to the criteria of Briggs et al.^12^ The respiratory subtype was diagnosed when at least 4 out of the following 5 respiratory symptoms were present during the panic attack: asphyxia or suffocation sensation; dyspnea; chest pain or discomfort; paresthesia; fear of dying.

At every visit (weekly), each patient received a diary in which they recorded any panic attack that occurred between the visits. The scales and measurements used for evaluating the clinical response were: 1) changes in the number of panic attacks in comparison with the baseline period; 2) the Global Clinical Impression scale and Global Patient's Impression scale scores for panic disorder; 3) the Hamilton anxiety scale.

### Statistical analysis

The age differences between the groups that received clonazepam and placebo were measured via Student's t test. The differences in panic disorder duration and the Global Clinical Impression scale baseline scores between the two groups were obtained using the Mann-Whitney test. To evaluate the differences between the clonazepam and placebo groups regarding the Hamilton baseline scores we used the Student t test. To evaluate the treatment response (changes in the number of panic attacks in comparison with the baseline period, changes in the Hamilton, Global Clinical Impression scale and Global Patient's Impression scale, we used Fisher's exact test. In the group that received clonazepam the differences obtained in the clinical responses of the respiratory and non-respiratory subtypes were measured using Fisher's exact test.

## RESULTS

The initial sample consisted of 34 panic disorder patients, 19 women and 15 men, with a mean age of 36.9 years (standard deviation, SD = ± 8.8). The median duration of the panic disorder in the sample was 24 months (percentile 25% = 4.0 months; percentile 75% = 48.0 months; range = 1.0 to 324.0 months).

The clonazepam group consisted of 20 patients, 13 female and 7 male, with a mean age of 35.9 years (SD = ± 8.6 years). The placebo group consisted of 14 patients, 6 female and 7 male, with a mean age of 38.3 years (SD = ± 9.3 years). There were no statistically significant age differences (t test, p = 0.447) or gender differences (Fischer exact test, p = 0.296) between the two groups.

The median duration of the panic disorder in the clonazepam group was 18 months (percentile 25% = 2.0 months; percentile 75% = 48.0 months; range = 1.0 to 264.0 months). In the placebo group the median duration of the panic disorder was 24 months (percentile 25% = 12.0 months; percentile 75% = 60.0 months; range = 3.0 to 324.0 months). With regard to the duration of the panic disorder there were no statistically significant differences between the two groups (Mann-Whitney test, p = 0.382).

There were no statistically significant differences between the two groups in the Global Clinical Impression scale and Hamilton anxiety scale scores in the baseline period. In the clonazepam and placebo groups the median scores for the Global Clinical Impression scale were respectively: 5.0 (percentile 25% = 4.0; percentile 75% = 5.0) and 5.0 (percentile 25% = 4.5; percentile 75% = 5.0), according to the Mann-Whitney test (p = 0.549). The mean scores for the Hamilton anxiety scale in the clonazepam and placebo groups were, according to the Student t test (p = 0.943), respectively: 26.1 (SD = ± 6.2) and 26.3 (SD = ± 8.2).

Of these 34 patients, one in the clonazepam group and one in the placebo group dropped out of the study, through missing visits. One woman in the clonazepam group was excluded from the study due to a major depressive episode and one woman in the placebo group was excluded due to the increase in anxiety and panic attack frequency. One male patient in the clonazepam group missed the second evaluation visit but remained in the study until the 6^th^ week. This patient was kept in the study because he missed just the second visit, but was present in the subsequent visits and kept clonazepam use until the 6^th^ week, when he was evaluated. The clonazepam group had a significant decrease in anxiety and panic attacks in comparison with the placebo group, especially in the 6^th^ week of the study, when we found remission of panic attacks (panic-free status) and clinical improvement, as shown through the Global Clinical Impression scale (0=normal; 1=almost normal; 2=mildly impaired) and Global Patient's Impression scale (1=much better; 2=better) and also through a 50% decrease in the Hamilton anxiety scale score (Table 1).

**Table 1:** Response to treatment in the clonazepam (C) and placebo (P) groups (n = 30) by panic disorder and agoraphobia patients

	C Group			P Group		C Group		P Group		
	Second week					Sixth week				
	Yes	No	Yes	No	p value	Yes	No	Yes	No	p value
Panic-free	8	9	2	10	* = 0.126	14	4	1	11	* < 0.001
GCI (0, 1 or 2)	10	7	3	9	* = 0.130	16	2	1	11	* < 0.001
GPI (1 or 2)	12	5	4	8	* = 0.067	15	3	4	8	* = 0.009
50% reduction in HAM	11	6	3	9	* = 0.060	14	4	4	8	* = 0.024

* *= Fisher exact test; GCI = global clinical impression; GPI = global patient's impression; HAM = Hamilton anxiety scale.*

In the clonazepam group (n = 18), 11 patients were classified in the respiratory subtype and 7 in the non-respiratory subtype of panic disorder. In the placebo group (n = 12), 6 patients were classified in the respiratory subtype and 6 in the non-respiratory subtype of panic disorder. In the clonazepam group there was no significant difference regarding the clinical efficacy of clonazepam between the respiratory and the non-respiratory subtype panic disorder patients, according to the clinical variables mentioned earlier (Table 2).

**Table 2:** Clonazepam Group patients' responses to this drug in respiratory and non-respiratory PD (panic disorder) subtypes

	Respiratory Subtype (n=11)		Non-Respiratory Subtype (n = 7)			Respiratory Subtype (n = 11)		Non-Respiratory Subtype (n = 7)		
Second week						Sixth week				
	Yes	No	Yes	No	p value	Yes	No	Yes	No	p value
Panic-free	6	4	2	5	* = 0.218	8	3	6	1	* 0.485
GCI (0, 1 or 2)	7	3	3	4	* = 0.268	9	2	7	0	* 0.359
GPI (1 or 2)	8	2	4	3	* = 0.314	9	2	6	1	* = 0.674
50% reduction in HAM	8	2	3	4	* = 0.145	8	3	6	1	* = 0.485

* *= Fisher exact test; GCI = global clinical impression; GPI = global patient's impression; HAM = Hamilton anxiety scale.*

## DISCUSSION

This is the first study that makes a comparison between two subtypes of panic disorder in relation to the clinical efficacy of clonazepam. We chose this drug due to its known efficacy in panic disorder treatment.

The results of this study suggest that the administration of clonazepam reduces panic attacks and anxiety in panic disorder patients. Clonazepam was superior to placebo in the efficacy parameters evaluated in this study (panic attack remission, favorable scores in the Global Clinical Impression scale and Global Patient's Impression scale and a 50% decrease in the Hamilton anxiety scale score). Although we had a small clinical sample in this study, the decrease in anxiety and panic attacks obtained with clonazepam were statistically significant in comparison with placebo.

Our results agree with others obtained earlier.^17,18^ One series of studies from the Massachusetts General Hospital-Harvard Medical School^10,19^ used clonazepam for over 400 patients with panic disorder. In a double-blind randomized study^19,20^ with clonazepam, alprazolam and placebo, 72 patients were treated for six weeks. At the end of this study both benzodiazepines were efficient and statistically superior to placebo regarding panic attack control, severity of phobias and incapacitation due to panic disorder. Both benzodiazepines were equally tolerated. The side effects (mainly sedation and ataxia) were mild, and a greater number of patients from the alprazolam group dropped out of the study.

Beauclair et al.,^21^ observed that 29 patients treated with clonazepam had a significant decrease in the frequency and severity of panic attacks in comparison with those treated with placebo. Judd and Burrows^22^ described an efficient treatment using clonazepam at between 1.0 and 8.0 mg/day in 10 patients with panic disorder and agoraphobia of varying severity. After treatment with clonazepam the frequency and severity of the panic attacks decreased significantly and sedation was the only side-effect observed.

Multicenter studies have also shown the efficacy of clonazepam efficacy in panic disorder treatment. Rosenbaum et al.,^23^ with a sample of 413 patients with panic disorder, found that clonazepam was superior to placebo regarding the decrease in the number of panic attacks, in anticipatory anxiety and phobic avoidance. Moroz and Rosenbaum^24^ in a study with 455 panic disorder patients found that clonazepam was superior to placebo regarding the remission of panic attacks and yielded a decrease of 50% in the score on the Hamilton anxiety scale.

According to DSM-IV,^16^ there are respiratory symptoms in panic disorder such as shortness of breath and smothering sensation or feeling of choking. Briggs et al.^12^ suggested that the physical symptoms of panic attacks in all patients may be similar, and appropriate differentiation based on their symptoms may be made via the presence or absence of respiratory symptoms. Valença et al.,^25^ in a phenomenological study of laboratory-induced panic attacks, found that the induction of panic attacks with the inhalation of 35% CO_2_ in a sample of 31 panic disorder patients produced more respiratory symptoms such as difficulty in breathing and suffocation/asphyxia sensation after CO_2_ inhalation. Biber and Alkin,^26^ in a study with similar methodology, used the 35% CO_2_ inhalation test to induce panic attacks in 51 panic disorder patients. These patients were separated into two subtypes: a "respiratory" subtype (n = 28), which had prominent respiratory symptoms, and a "non-respiratory" subtype (n = 23). These authors observed that 22 (79%) of the 28 patients in the "respiratory" subtype and 11 (48%) of the 23 patients in the "non-respiratory" subtype had panic attacks after the 35% CO_2_ inhalation (a statistically significant difference). They raised the hypothesis that patients with the "respiratory" subtype of panic disorder could have an increased panic response when submitted to CO_2_ inhalation.

Bandelow et al.,^14^ found that a group of panic disorder patients with cardiorespiratory symptoms (fear of dying, chest pain, shortness of breath, paresthesia and smothering sensation) had less frequent situational panic attacks and more frequent spontaneous panic attacks. Biber and Alkin^26^ pointed to the fact that patients with prominent respiratory symptoms had more spontaneous and nocturnal panic attacks; a previous history of smothering traumatic events; a previous history of respiratory diseases; a history of heavy smoking; increased panic disorder duration; and better clinical response to tricyclic antidepressant treatments.

Some studies have tried to associate panic disorder subtypes and clinical responses to different classes of psychopharmacological therapies. Mavissakalian^27^ observed that respiratory symptoms had a favorable response to imipramine and could be used as early clinical response markers, in a study using imipramine and placebo in 63 panic disorder patients. Briggs et al.^12^ found that patients with prominent respiratory symptoms had better responses to imipramine, and patients in the "non-respiratory" subtype had better responses to alprazolam and more situational panic attacks, in a study with a sample of more than one thousand panic disorder patients.

In our study we did not find significant differences regarding the clinical response to clonazepam when we compared the respiratory and non-respiratory subtypes of panic disorder. Differing from preceding studies^12,27^ that showed better therapeutic response to imipramine among panic disorder patients with the respiratory subtype, we found that panic disorder patients in the respiratory subtype and the non-respiratory subtype had favorable responses to clonazepam at a dosage of 2 mg/day, with panic attack remission and decrease in anxiety in both subgroups.

## CONCLUSION

Clonazepam was equally effective in the treatment of panic disorder patients with the respiratory and non-respiratory subtypes. This study confirms the evidence of the efficacy of clonazepam in panic disorder treatment. Although we had a small sample, our results are supported by the evidence from the literature regarding the therapeutic effects of clonazepam in the treatment of panic disorder.
